# Epigenetic Effects of Benzene in Hematologic Neoplasms: The Altered Gene Expression

**DOI:** 10.3390/cancers13102392

**Published:** 2021-05-14

**Authors:** Giovanna Spatari, Alessandro Allegra, Mariella Carrieri, Giovanni Pioggia, Sebastiano Gangemi

**Affiliations:** 1 Department of Biomedical and Dentistry Sciences and Morphological and Functional Imaging, University of Messina, 98125 Messina, Italy; giovanna.spatari@unime.it; 2 Division of Hematology, Department of Human Pathology in Adulthood and Childhood “Gaetano Barresi”, University of Messina, 98125 Messina, Italy; aallegra@unime.it; 3 Department of Cardiac Thoracic Vascular Sciences and Public Health, University of Padova, 35128 Padova, Italy; 4 Institute for Biomedical Research and Innovation (IRIB), National Research Council of Italy (CNR), 98164 Messina, Italy; giovanni.pioggia@cnr.it; 5 School of Allergy and Clinical Immunology, Department of Clinical and Experimental Medicine, University of Messina, 98125 Messina, Italy; sebastiano.gangemi@unime.it

**Keywords:** benzene, epigenetic, leukemia, lymphoma, hematological malignancies, gene expression, cancer, air pollution, occupational disease

## Abstract

**Simple Summary:**

Benzene is produced by diverse petroleum transformation processes and it is widely employed in industry despite its oncogenic effects. In fact, occupational exposure to benzene may cause hematopoietic malignancy. The leukemogenic action of benzene is particularly complex. Possible processes of onset of hematological malignancies have been recognized as a genotoxic action and the provocation of immunosuppression. However, benzene can induce modifications that do not involve alterations in the DNA sequence, the so-called epigenetics changes. Acquired epigenetic modification may also induce leukemogenesis, as benzene may alter nuclear receptors, and cause changes at the protein level, thereby modifying the function of regulatory proteins, including oncoproteins and tumor suppressor proteins.

**Abstract:**

Benzene carcinogenic ability has been reported, and chronic exposure to benzene can be one of the risk elements for solid cancers and hematological neoplasms. Benzene is acknowledged as a myelotoxin, and it is able to augment the risk for the onset of acute myeloid leukemia, myelodysplastic syndromes, aplastic anemia, and lymphomas. Possible mechanisms of benzene initiation of hematological tumors have been identified, as a genotoxic effect, an action on oxidative stress and inflammation and the provocation of immunosuppression. However, it is becoming evident that genetic alterations and the other causes are insufficient to fully justify several phenomena that influence the onset of hematologic malignancies. Acquired epigenetic alterations may participate with benzene leukemogenesis, as benzene may affect nuclear receptors, and provoke post-translational alterations at the protein level, thereby touching the function of regulatory proteins, comprising oncoproteins and tumor suppressor proteins. DNA hypomethylation correlates with stimulation of oncogenes, while the hypermethylation of CpG islands in promoter regions of specific tumor suppressor genes inhibits their transcription and stimulates the onset of tumors. The discovery of the systems of epigenetic induction of benzene-caused hematological tumors has allowed the possibility to operate with pharmacological interventions able of stopping or overturning the negative effects of benzene.

## 1. Introduction

### 1.1. General Considerations on Benzene and Neoplasms

Benzene is a ubiquitous environmental contaminant (air, soil, water), classified in group 1 by the International Agency for research on cancer [1]. It comes from both natural, such as volcanoes and forest fires, and anthropogenic sources that include combustible fuel emissions, vehicles exhaust, hazardous waste sites or industry. Benzene is produced by different petroleum conversion processes and used as an intermediate in the production of a wide number of chemical substances or in the manufacturing of plastics, nylon and synthetic fibres, rubber detergents and pesticide [2].

The main sources of benzene exposure for the general population include vehicle exhaust and cigarette smoke [3,4], while considerably lower exposures to benzene can occur from consumption of food, water and beverages [5]. The limitation of the benzene content in gasoline in 1998 by EU Directive 98/70/EC and the prohibition to smoke in many public places reduced the benzene exposure of the general population significantly. Moreover, in order to improve air quality in Europe, a limit value for the protection of human health of 5 µg/m^3^ (0.0015 ppm) has been set by the European Directive 2008/50/EC on ambient air quality and cleaner air for Europe of the European Parliament and of the Council.

Occupational exposure to benzene occurs in the petroleum and chemical industries and also as a result of exposure to gasoline engine emissions and combustion products. In occupational settings, in the past, benzene exposure was high with estimated concentrations in the range 10–100 ppm or even higher [6]. Recent studies showed that the occupational exposures to benzene in Europe decreased and are usually below 0.1 ppm, although exposures above 0.1 ppm have been reported for some tasks such as gasoline pump repair and maintenance, or for fuel-tanker drivers or work in refineries [7,8,9,10,11,12].

### 1.2. Absorption and Metabolism of Benzene

Inhalation is the principal route of exposure to benzene; oral or dermal exposure is also possible although the uptake is small compared to via inhalation [13].

Assessing the cutaneous exposure to benzene, an essential element is percutaneous assimilation, and two factors are critical in absorption evaluations: the rate of benzene absorbed and the permeability coefficient (Kp). The skin has a lipophilic edge, the stratum corneum (SC), while the epidermis and dermis, which are hydrophilic, are beneath. To arrive in the blood, benzene must go by the epidermal cells, the aqueous pores, or the skin appendages. After this, a system of capillaries permits the absorption of the substance. A different modality to pass the SC edge is available intracellularly. Diffusion across this barrier establishes the superior limit of the dermal penetration coefficients for benzene, with a Kp of about 0.1 cm/h [14].

The percutaneous absorption of benzene was evaluated by employing 14 C-benzene at a dosage of 100 μL on the forearm. This gave rise to a benzene dose absorption value of 0.065% ± 0.042%, suggesting a small amount of percutaneous absorption [15]. In fact, with respect to absorption from inhalational uptake, percutaneous uptake is insignificant, with the dermal/inhalational proportion probably being <4 [16].

Analyses of the inhalational absorption of benzene report mean absorption percentages oscillating from about 50 to 80%. Benzene, after absorption, is quickly scattered in all tissues, and the substance has been found in different organs, in biological fluids and in the placenta [17]. Benzene allocation may be governed by the perfusion degree of tissues, with greater amounts in organs such as the lung, brain, kidney, and spleen [17].

**Figure 1:**
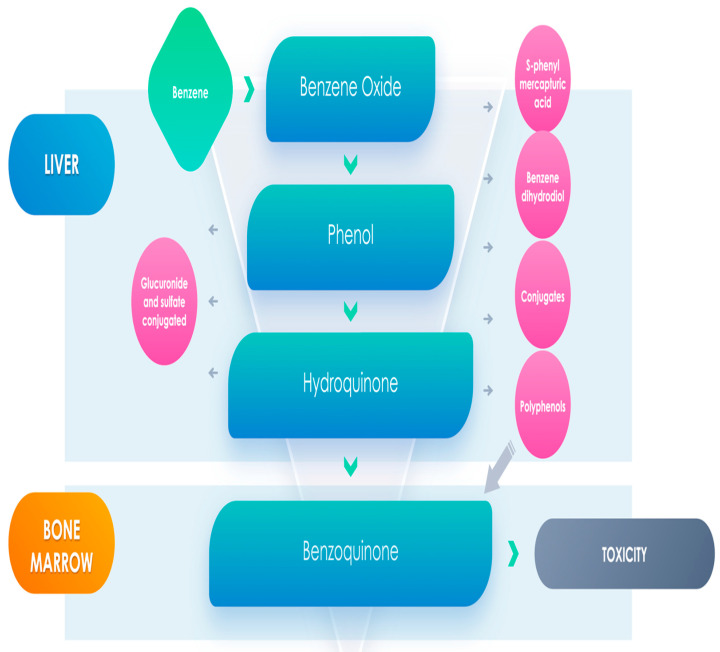
Benzene metabolites could have a main role in leukemogenesis.

As far the metabolism of benzene, it happens essentially in the liver but also in the lung, with a subsequent metabolism taking place in the bone marrow. It begins with oxidation to benzene oxide principally by cytochrome P450 2E1, and this substance rapidly is transformed to phenol, and then into catechol and hydroquinone metabolites, both of which can be commuted into noxious elements [18]. Otherwise, benzene oxide may be transformed to benzene dihydrodiol, which can be changed to catechol [17,18], and there is also the possibility for the generation of aldehyde metabolites. Benzene metabolites may gather in the bone marrow where heme-protein peroxidases activate phenolic metabolites to semiquinone radicals generating reactive oxygen species, causing further damage to bone marrow cells, which could be essential for the determinism of hematological diseases [19] (Figure 1).

Some analyses have essentially studied the percentage of benzene elimination expired in air and through phenols in the urine. Exhalation is the principal way for elimination of unmetabolized benzene, while benzene is eliminated essentially through urine [20]. The amount of benzene found in expired air drops quickly, but in spite of this, residues are still present after 24 h. Phenol removal via the urine starts about 2 h after exposure, with the phenylsulfate discovered until its urine amount is 400 mg/L, after which phenylglucuronide is also excreted [21].

Moreover, the intricacy of benzene’s metabolic characteristic is augmented by genetic polymorphisms that have been described for enzymes implicated in benzene metabolism such as CYP2E1, glutathione-*S*-transferases (GST) GSTM1, GSTT1, GSTP1, and NAD(P)H:quinone oxidoreductase 1, which may modify the metabolism of benzene [22]. Finally, a research has demonstrated that variations in polymorphic gene frequencies occur between Africans, Caucasians, and Asians, principally concerning GSTM1, GSTT1, and GSTA1 [23].

### 1.3. Benzene and Cancer

Several studies have demonstrated that exposure to benzene is capable to cause the onset of solid neoplasms such as breast cancer and urothelial carcinoma. A case-control research by Petralia et al. [24] and other works indicated a correlation between breast cancer and occupational contact with benzene [24,25]. Moreover, Costantini et al. [26] performed an epidemiological cohort study of female workers employing benzene-based glues in a shoe industry in Italy. The analysis confirmed that chronic exposure to benzene can be one of the risk elements for breast cancer [26,27,28], while in a population-based case-referent study of urothelial cancer in Stockholm an exposure to benzene gave an increased relative risk of 2.0 [29,30].

### 1.4. Benzene and Hematological Malignancies

A larger amount of data is present in the literature to affirm a pathogenetic correlation between exposure to benzene and the onset of hematological neoplasms.

A study, carried out on shoe manufacturers exposed to benzene over a 16-month period, showed that exposure to benzene ≤1 ppm could still provoke injury to the human organism and to the hematopoietic system [31]. The workers were grouped according to their exposure in three groups (<1, 1–10 ppm, >10 ppm. over a 1-month monitoring period). The hematological evaluations conducted on the subjects showed that in the lowest exposure group, leukocyte and platelet counts were significantly decreased relative to the control values (8–15% lower). In the highest exposure group, the decrement was higher. However, a subsequent evaluation of the same data conducted by Lamm & Grunwald [32] concluded that while hematotoxicity was demonstrated at benzene concentrations greater than 10 ppm, it was insubstantial at lower concentrations.

A systematic review recognized 16 reports, which jointly evaluated the occurrence of hematological malignancies across 187,585 residents residing close to a refinery. Inhabitants from the areas less than 5 km from a petrochemical facility presented a 30% higher risk of developing hematological malignancies than habitants from areas with no petrochemical activity [33].

### 1.5. Benzene and Leukemia

Benzene is acknowledged as a myelotoxin with leukemogenic effects [34]. In fact, it is widely considered as an example of environmental leukemogenic with chronic contact correlated with an augmented risk for acute myeloid leukemia (AML) [35,36,37].

AMLs are a set of hematological tumors that implicate clonal growth of immature myeloid progenitor cells in the bone marrow and peripheral blood. These blast cells are disposed to be extremely proliferative and can alter normal hemopoiesis. Their growth causes a mass of non-functional cells, inducing neutropenia, anemia and thrombocytopenia.

Exposure to elevated levels of benzene (>100 ppm) causes damage in the hemopoietic system, and other forms of leukemia than AML have also been reported [38].

Numerous investigations were performed by the Chinese Academy of Preventive Medicine (CAPM) and the US National Cancer Institute (NCI) on a large number of workers exposed to benzene or benzene-containing combinations. The NCICAPM analyses proved an augmented risk of AML as well as other malignant hematopoietic disorders, such as myelodysplastic syndromes (MDS), a group of clonal hematopoietic stem cell diseases distinguished by ineffective hematopoiesis, peripheral cytopenia, dysplasia in the bone marrow, and an augmented risk of evolving to AML, occurring in about 30% of the patients.

However, in contrast to the above, these results stated an increased risk at concentrations of benzene exposure lower than 10 ppm as average and lower than 40 ppm-years cumulative [39,40,41]. In fact, a nested case-control report was also performed in Australia employing the Australian petroleum industry-monitoring data [42]. The study stated an augmented risk of leukemia correlated with total benzene exposure lower than before reported; for instance, a total exposure above 8 ppm-years augmented the risk for acute nonlymphocytic leukemia by 7-fold [43,44]. Exposure to benzene also provokes diverse damages to the hematopoietic system, comprising variable grades of pancytopenia and aplastic anemia [45,46,47].

### 1.6. Benzene and Lymphoproliferative Diseases

Other forms of malignancy, such as lymphoproliferative malignancies, were also correlated with chronic exposure to benzene.

Lymphomas are a composite group of tumors—neoplasms of the hematopoietic system, distinguished by the abnormal growth of lymphoid cells or their precursors. Generally, lymphoma is classified into two different groups: non-Hodgkin’s lymphoma (NHL, 90%) and Hodgkin’s lymphoma (HL, 10%). However, the multiplicity of these diseases and the infrequency of some types of the tumors have made it problematic to evaluate the hazard in epidemiological analyses [48]. A report by Glass et al. displayed a correlation between benzene exposure with chronic lymphocytic leukemia (CLL) [42], while the NCI-CAPM analysis identified a 3-fold augment in risk for NHL among the benzene exposed employers, with risk augmenting to 4-fold for employers with 10 or more years of benzene exposure [49]. Meta-analyses of reports on NHL and benzene exposure in refineries and manufacturing other than refineries suggested that both benzene exposure and refinery work were correlated with augmented risks of NHL.

Multiple myeloma (MM) is a plasma neoplasm characterized by an aberrant proliferation of clonal, terminally differentiated B lymphocytes, and a few reports have also correlated MM with benzene exposure [50,51,52].

Acute lymphoblastic leukemia (ALL) is the most frequent type of childhood tumor and accounts for about 20% of all pediatric malignancies. The present survival percentage for pediatric ALL has enhanced (>90%) recently. However, about 20% of children with ALL will finally go through relapse, and the prognosis of relapse is disappointing.

In recent times, a correlation of childhood ALL with air pollution sources, such as gas stations, and automobile repair garages that produce benzene, was proposed [53,54]. The discovery that childhood leukemias are possibly started in utero sustains the idea that exposure of the mother to benzene is also relevant in their onset and progression [55,56]. In fact, experimental animal models demonstrated that in utero exposure to benzene augmented micronuclei occurrence and DNA recombination episodes in fetal and postnatal hematopoietic tissues [57,58].

### 1.7. Benzene and Stem Cells

As all hematopoietic tumors result from altered stem cells, some authors suggested that different types of myeloid and lymphoid tumors, comprising their pre-stages, can be provoked by occupational exposure to benzene [59]. Benzene and its metabolites cause harmfulness toward hemopoietic stem cells (HSCs), comprising short- and long-lasting injury to HSCs, and determine negative effects on the BM hematopoietic milieu [60]. Benzene contact decreases not only the rate and amount of BM HSCs in animals, but also reduces colony generation, which was also reported in in vivo analyses [61,62].

Yoon et al. described that the HSCs cell cycle was inhibited by p53-regulated increase of p21 in animals subjected to 300 ppm of benzene for 2 weeks [63]. Additionally, permanent DNA injury was reported in Lin- C-kit+ Sca-1+ cells of mouse, 8 months after benzene exposure [64], and benzene metabolites have analogous noxious actions on HSCs. It was also reported that 5 μM of 1,4-benzoquinone extremely blocked the proliferation of single-lineage progenitor colonies such as colony-forming unit-erythroid, -granulocyte or -monocyte, and burst-forming unit-erythroid (BFU-E), while greater dosages of 1,4-benzoquinone (7 and 12 μM) remarkably decreased colony amounts of multi-lineage (progenitor colonies such as colony-forming unit- granulocyte–macrophage and colony-forming unit-multipotential myeloid stem cell) in animal HSCs in vitro [65].

In adjunct to harmfulness toward HSCs, benzene and its metabolites are toxic to the BM niche, which is a specific milieu containing diverse hematopoietic stem cells, stromal cells, immune cells, matrix, and cytokines [66]. Rivedal et al. demonstrated that two metabolites of benzene (trans-muconaldehyde and glutaraldehyde) were the main factors related to the blocking of gap junction intercellular communication [67]. Moreover, the oxidation of HQ into 1,4-benzoquinone causes disproportionate oxidative damage to stromal cells, altering their capacity to produce Nicotinamide adenine dinucleotide phosphate (NADPH) quinone oxidoreductase 1, and decreasing CD34 + cell adhesion [68].

Probably, the effects of benzene on the BM niche could be the main culprits for hematological neoplasms related to exposure to the substance.

## 2. Mechanisms of Benzene Carcinogenesis

The type of manners by which pollutants participate to carcinogenesis can be multiple. However, these processes can be classified into a restricted number of cases. Guyton et al. reported diverse groups of key events correlated with carcinogens that exemplified several carcinogenic processes [69]. Carcinogens generally exhibit ≥1 of the 10 principal characteristics such as: be genotoxic; cause an alteration of DNA repair systems or provoke genomic instability; operate as an electrophile either directly or after metabolic activation; cause oxidative stress and chronic inflammation; be immunosuppressive; control receptor mediated actions; induce immortalization; modify cell growth, cell death, or nutrient supply; and cause epigenetic alterations [70]. Some of these effects are likely to occur in humans at low exposure levels to benzene (≤1 ppm), in particular genotoxicity (clastogenicity and aneugenicity), immunotoxicity, altered gene expression, and receptor-mediated effects.

Several factors have been implicated to explain the onset of benzene-caused hematological neoplasia, such as activation of benzene in the liver to phenolic metabolites with the subsequent transport of these substances to the bone marrow and transformation to semiquinone radicals and quinones through peroxidase enzymes (Figure 1). Other causes might be the generation of active oxygen species through redox cycling or the alteration of DNA or DNA associated protein, tubulin, or topoisomerase.

From all this, it is clear that the leukemogenic mechanism of benzene is certainly much more complex than what was reported so far. A possible mechanism of initiation of tumors was identified as an effect on oxidative stress and inflammation and the provocation of immunosuppression.

Elements of the oxidative stress and inflammatory pathways, such as Tumor Necrosis Factor (TNF)-α, Interleukin (IL)-6, and superoxide dismutase-1 (SOD1), are modified after benzene exposure, even at concentration in the range 0.5–5 ppm [71]. Moreover, glutathione transferases (GSTs) are important mediators in oxidative stress responses, and the metabolic genotype of GST seems able to modulate the metabolism of benzene [72].

Experimental models have proposed an empowering effect: in double combinations, the toluene–benzene mixture in animals has displayed a synergistic, haemato-toxic action [73]. Analyses of exposure to three-components combination (benzene, toluene, and xylene) in cell lines have demonstrated a direct action on programmed cell death resulting from reactive oxygen species (ROS) generation [74].

### Benzene and Immune System

Regarding the benzene immunosuppressive effect, results of a meta-analysis propose a benzene-caused action on the adaptive immune system and stimulation of the innate immune system. Benzene considerably reduces the number of white blood cells, especially CD4+ T-cells, B-cells and natural killer cells [75]. Particularly interesting is the existence of the positive relationship between age and serum levels of IL-10 [76], and a clear involvement of the cytokine network [77]. This agreed with the more potently suppressed production of anti-inflammatory cytokine IL-10 from CD4(+) and CD25(+) T-cells in the elderly individual more than in the young individual [78].

However, it appears that there are other most important mechanisms of the leukemogenic effect of benzene. The actions that commence leukemogenesis seem to be correlated to DNA damage. It is well-known that an approximate 45–55% of AML patients have sign of a chromosomal alteration and 95–96% have demonstrable genomic alterations. Probably, 100% of patients have such modifications, some of which have not yet been recognized [79].

Regarding the mutagenicity of benzene, some studies suggest clastogenic and aneugenic effects at concentrations below 1 ppm in oil refinery workers [80,81]. For concentrations from 0.1 to <1 ppm, the effects are borderline or absent [10,82], while for concentrations below 0.1 ppm, no relevant effects are reported [7,83,84].

The benzene metabolites 1,2,4-benzenetriol and hydroquinone (HQ) are genotoxic and provoke chromosomal alterations which promote the onset of leukemias [85,86,87]. In vitro exposure to HQ at small levels such as 0.2–2.5 μM over 20–72 h provoke monosomy 7 in human bone marrow (BM) and cord blood CD34 + progenitor cells [88]. Selective deletion of chromosome 5q31 at levels as low as 2.5 μM has been reported in human BM CD34 + cells [89].

The DNA damage comprises DNA strand breakage, mitotic recombination, chromosome translocations, and aneuploidy. If these events happen in stem cells, a leukemic clone with specific vantage to proliferate may result, as a consequence of pro-oncogene stimulation, or suppressor gene deactivation [90].

The theory that the genotoxic actions of benzene are subsequent to oxidative stress and inhibition of topoisomerase II was verified by mechanism-oriented transcriptomics studies in p53-knockout and wild-type animals [91]. In vivo and in vitro tests for chromosome abnormalities confirmed the occurrence of these aberrations after benzene exposure. Moreover, experimentations gave essential data to explain the mechanisms implicated in benzene genotoxic capacity, comprising its ability to provoke aneuploidy. However, the findings attained from the in vivo gene mutation tests stated the modest suggestion for benzene’s potential to cause gene mutations. This possibility is very low and is only reported when employing great cumulative dosages for a long time [92].

**Figure 2:**
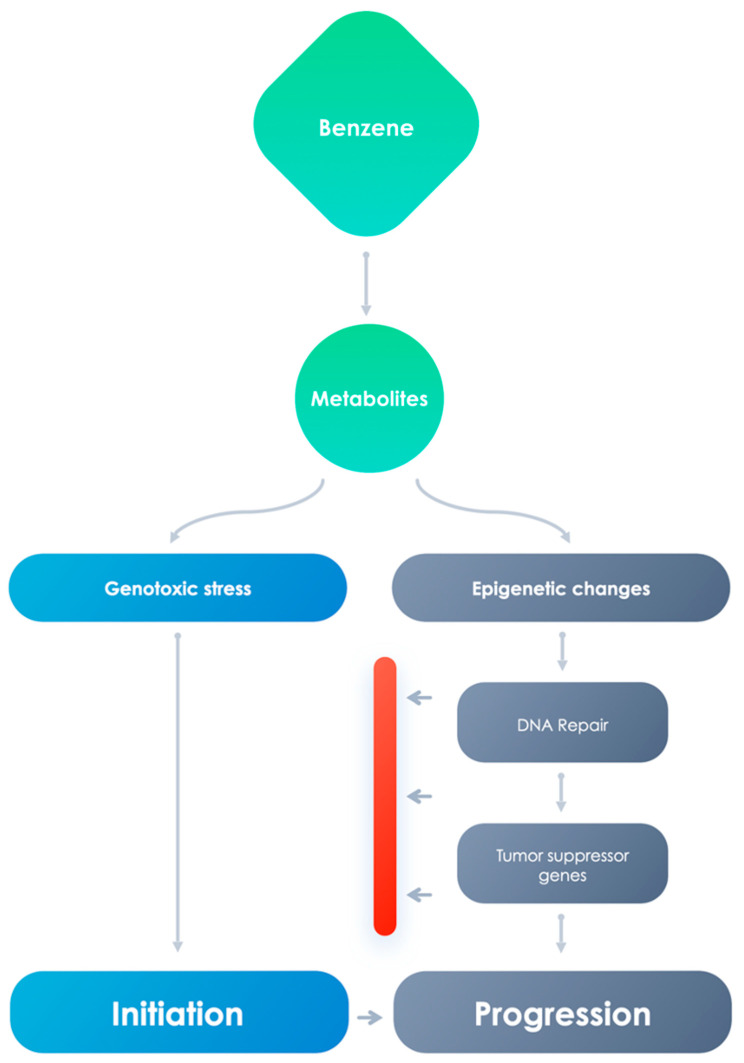
Genotoxic effects and epigenetic changes can influence carcinogenetic development.

However, it is becoming evident that genetic alterations are insufficient to fully justify several phenomena that influence the onset of leukemias. For example, monozygotic twin pairs, in spite of the presence of indistinguishable DNA sequences, are often different for several characters, suggesting that the identical genotype can have different phenotypes. Although the onset and development of AML after benzene exposure is usually supposed to be analogous to therapy-related AML, in which clonal cytogenetic alterations are believed to be starting events [93,94], nevertheless, this statement is not sustained by quantitative analyses describing cytogenetic studies for actual disease outcomes. One epidemiologic report tried to associate cytogenetic alterations with hematopoietic tumors, the results were not distinctive with respect to pollutant exposure or disease [95]. This indicates the participation of supplementary elements that cannot be clarified exclusively by the genomic and propose the systems via which benzene is able to induce carcinogenesis are many, both genotoxic and non-genotoxic (Figure 2).

## 3. Epigenetic and Cancer

Human DNA is strictly packed into chromatin by enveloping the core histone octamer of nucleosomes. The N- and C-termini, projecting out from the nucleosome, are expose to a broad assortment of post-translational amino acid alterations comprising methylation, acetylation, phosphorylation, ubiquitinoylation, and SUMOlytion. These alterations of the histones, combined with DNA alterations, act in a synergistic manner to control gene stimulation or silencing in chromatin. The ε-N-acetylation of lysine counterbalances positive charges on histones, diminishing their relations with negatively charged DNA and acts in transmuting chromatin into a relaxed condition and activating the gene transcriptional apparatus for gene activation [96].

In physiological situations, it has been evaluated that methylated cytosines correspond to 2–6% of all the cytosines in normal cells [97]. DNA methylation regulates several biologic activities comprising the gene expression, embryonic growth, X-inactivation, cellular differentiation, silencing of transposable components, and genomic imprinting [98,99].

The word “epigenetic” indicates longstanding alterations in chromatin structure and gene expression that are not provoked by modifications in the DNA sequence itself and can be heritable over cell divisions [100]. Epigenetic events can influence the carcinogenic development by disturbing gene expression and DNA reparation systems [101]. A broad variety of carcinogens have been reported to alter the epigenome, and it has been suggested that their mechanism of action may involve disruption of epigenetic mechanisms [101].

DNA hypomethylation correlates to stimulation of oncogenes [102], while the hypermethylation of 51 cytosine-phospho-guanine (CpG) islands in promoter regions of specific tumor suppressor genes inhibits their transcription and stimulates the onset and the progression of tumors [103].

Chappell et al. [104] performed a systematic analysis of the paper on carcinogens that presented epigenetic endpoints. They evaluated pollutants classified as “carcinogenic to humans” (Group 1), comprising benzene, with convincing suggestion of genotoxic and non-genotoxic mechanisms of carcinogenesis. They evaluated 158 papers that studied epigenetic alterations for 12 carcinogenic elements comprising benzene. 10 or more works stated epigenetic effects [104].

Non-genotoxic carcinogens may affect nuclear receptors, and provoke post-translational alterations at the protein level, so touching the stability or function of regulatory proteins, comprising oncoproteins and tumor suppressor proteins [105]. Changed epigenetic condition provokes genome instability and alteration of controlled proliferation signs, habitually detected in tumor cells [106,107,108].

## 4. Epigenetics, Hematological Neoplasms, and Benzene

In addition to genotoxic modifications, acquired epigenetic alterations may participate in benzene leukemogenesis [109,110,111], and numerous findings are more coherent with an epigenetic model for onset of benzene-AML in which modified homeostatic control in the BM niche, not cytogenetic damage, prevails in the initial elaboration of the leukemic stem cell phenotype, a system completely different from preceding models of clonal cytogenetic damage [112].

As reported above, a modified DNA and histone methylation process could be implicated in leukemogenesis. Benzene could act modifying histones, globular proteins that undergo posttranslational modifications that modify their correlation with the DNA and other nuclear proteins. H3 and H4 histones have long tails protruding from the nucleosome, which can be covalently modified by acetylation, methylation, ubiquitination, phosphorylation, SUMOylating, citrullination, and ADP-ribosylation, and thus change chromatin structure and gene expression.

DNA methylation is determined by three DNA methyl-transferases (DNMTs) [113]; abnormal concentrations of DNMTs caused by pollutant carcinogens have been already reported all through the leukemogenesis [114].

Histone methylation happens on all basic residues and can occur at diverse residues on the same histone performed by numerous histone methyl-transferases, although certain histone modifications are reciprocally exclusive [115]. The most explored combined methylation is the specific feature that unites the activating H3K4me3 and the repressive H3K27 tri-methylation (me3). This situation was first suggested as a system to silence developmental genes while maintaining them ready for activation and is absent after cell differentiation [116]. Currently, this condition is recognized to also be existing in tumor cells, where it can cause both inhibition of until that time active genes (after loss of H3K4me3), or stimulation of beforehand inhibited genes correlated with tumor progression (after loss of H3K27me3) [117,118].

**Figure 3:**
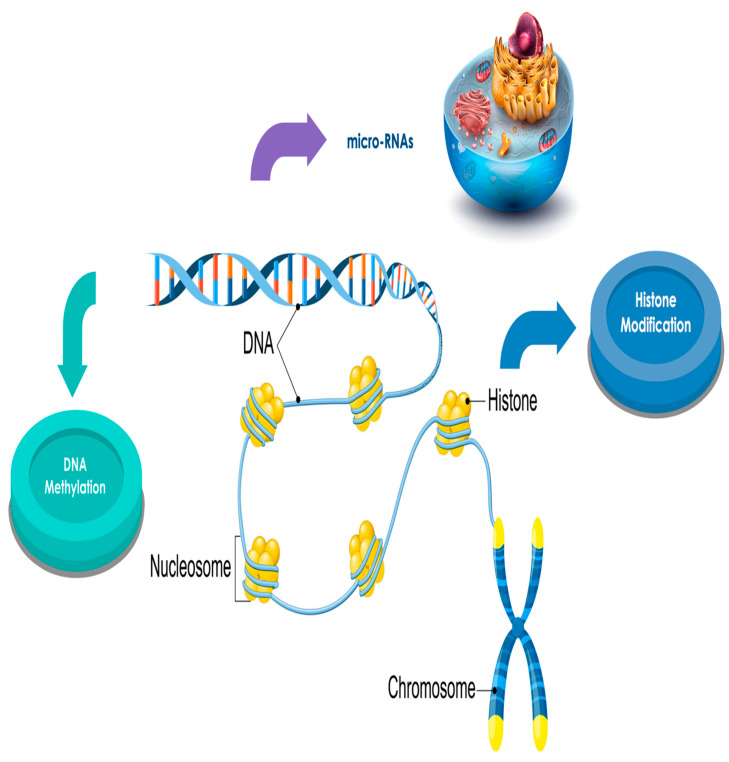
Benzene acts regulating histone methylation to change chromatin stratus.

Furthermore, DNA methylation has an action in controlling histone methylation, and vice-versa, with these two conditions sustaining each other to determine the chromatin status [119,120] (Figure 3).

Abnormal DNA methylation configurations, comprising gene-specific hypomethylation or hypermethylation, total hypomethylation, and loss of imprinting (LOI), are usual in AML. Total genomic DNA methylation amounts tend to reduce in each phase of the evolution from normal to AML cells [121]. As far the gene-specific hypomethylation, a reduced methylation of melanoma-associated antigens-1 (MAGE-1), a gene hypomethylated in leukemic cells, has been reported [122,123] (Table 1). Moreover, DNA methylation is accountable for imprinting in mammal cells, and this induces certain genes to be expressed either by the paternally or maternally inherited chromosome. LOI is an initial epigenetic occurrence in leukemogenesis that was recognized in blood from patients with AML and MDS but not in peripheral cells or BM hematopoietic progenitor cells from normal subjects [124].

A particular epigenetic role could be played by benzene in particular forms of leukemia. Children acute leukemia exhibits distinctive clinical and biological characteristics and is generally accompanying to alterations in the mixed-lineage leukemia (MLL) gene (MLL-r), a gene positioned on chromosome 11q23 that controls physiological hematopoietic expansion and differentiation [125]. The MLL-r codes a methyltransferase with action on lysine 4 of histone H3 (H3K4), which, as reported above, provokes modifications in chromatin correlated with epigenetic transcriptional stimulation with activation of gene expression during hematopoiesis [126]; interesting changes to the MLL gene have been described only in animals with alterations in DNA damage response but not in wild-type mice [127]. MLL-r operates as the starting oncogenic occurrence by altering epigenetic activities [102], and genetic and epidemiological analyses have demonstrated that MLL-r may origin from transplacental exposure to benzene metabolites (i.e., benzoquinone) during pregnancy [128,129]. Interestingly, and contrary to the generally accepted dogma of tumor biology, MLL-r children leukemia has been reported to have aberrant hypermethylation in non-enhancer, non-promoter regions [130,131,132,133,134].

### Benzene and Hemopoietic System: In Vitro and In Vivo Studies

As it concerns the epigenetic effects exerted by benzene on hematopoietic cells, 1514 differentially expressed genes (DEGs) in BM HSCs and 1703 DEGs in Peripheral BSCs (PBSCs) were evaluated in male C57B/6 animals exposed to benzene. Weighted gene correlation network analysis revealed that transcriptional alterations in hematopoietic cell lineage are essential pathways implicated in benzene-caused damage in BM HSCs. Interestingly, there were 164 common DEGs in both BM and peripheral HSCs, out of which 53 genes were co-controlled in both types of HSCs. Successive pathway investigation of these 53 genes suggested that the most important pathways implicated neutrophil degranulation and CD93 contained in the core of the network of the 53 genes, which are recognized to control leukemia stem cell quiescence and self-renewal [135].

Bollati et al. evaluated if epigenetic alterations occurred in normal subjects by low-level exposure to benzene [123]. Blood DNA samples and exposure information were achieved from traffic police officers, gas station employers, and unexposed subjects (benzene range, <6–478 Mg/m3). Bisulfite-PCR pyrosequencing was employed to quantify DNA methylation in long interspersed nuclear element-1 (LINE-1) and AluI repetitive elements as a substitute of genome-wide methylation and evaluate gene methylation of MAGE-1 and p15. Benzene was correlated with a relevant decrease in LINE-1 and AluI methylation. Increased methylation in p15 and reduced methylation in MAGE-1 were correlated with augmented airborne benzene concentrations. LOI was evidenced only in exposed subjects and not in referents [136].

Analogous findings were reported in a different experimentation in which DNA methylation status after exposure to a VOC mixture comprising benzene and ethylbenzene was evaluated. The analysis was performed on samples from gas station employers (GS) and leather shoe manufacturing workers (LS). SOD1, DNA topoisomerase 2-alpha (TOP2A), and TNF-α promoter methylation condition was augmented in LS. Moreover, in LS, authors also displayed important connection between glutathione S-transferase p1 gene (GSTP1) promoter methylation and both inducible Nitric oxide synthases (iNOS) and cyclooxygenase-2 **(**COX-2) methylation. In exposed subjects, ethylbenzene exposure amounts demonstrated a relevant connection with TOP2A methylation. These intracellular alterations may be the early system of toxicity causing hematopoietic tumors, perhaps due to a synergistic action of VOC combination [137].

In a different study authors evaluated if long-lasting exposure to low dosages of HQ might be adequate to modify in vitro the epigenetic profile [138]. In HL-60 cell, a human leukemia cell line, investigating the epigenetic alterations happening in chromatin, they demonstrated the temporary occurrence of a specific mark linking the repressive H3Lys27 tri-methylation signature and the stimulating H3Lys4 tri-methylation signature (H3K27me3/H3K4me3), suggesting a trend toward a balanced chromatin conformation. These modifications are missing in time after short-term exposure, while the long-lasting setting displayed a continuing rise in H3K4me3, indicating that prolonged treatment could provoke permanent epigenetic modifications [138]. Benzene also provoked total DNA hypomethylation in human lymphoblastoid TK6 cells at amounts of 1–100 μM [139].

However, diverse results were obtained in other experimentations. No relevant total DNA methylation modifications were reported in a work employing normal hepatic L02 cells or human myeloid HL-60 cells that were treated with benzene for 48 h and which presented alterations in gene expression amounts [140,141], although the exposure levels evaluated were analogous or even greater than those employed in the previous experimentation [139]. Further studies are needed to justify these discrepancies.

Several reports have been performed to investigate the chance that benzene can interfere in the onset of hematological tumors by operating on the tumor suppressor genes. Situated at 9p21 gene cluster region, *p14ARF* and *p15INK4b* anti-oncogenes have essential actions in controlling cell growth [142]. This renders this gene cluster an objective for selective block during carcinogenic process [143]. In fact, elevated accumulations of CpG islands on promoter regions of these genes disposes them to frequently be deactivated by promoter methylation [144,145]. Deactivation of *p14ARF* and *p15INK4b* genes by changed promoter methylation has been reported in several forms of neoplasms [146].

Jamebozorgi et al. evaluated whether chronic work-related exposure to a small amount of benzene is correlated with the methylation of the *p14ARF* and *p15INK4b* promoter CpG islands [147]. Total DNA methylation and promoter-specific methylation of the two tumor suppressor genes, *p14ARF* and *p15INK4b*, were performed employing the DNA derived from 40 petrochemical employers subjected to ambient benzene concentrations of <1 ppm, and 31 office employers not subjected to benzene. A rise in total DNA methylation of 5% in *p14ARF* and 28% in *p15INK4b* genes was demonstrated in the exposed subjects, while no augmented methylation in the considered genes was detected in the unexposed controls [147].

This result confirms that long lasting work-related exposure to levels inferior to the approved exposure limit of benzene may still provoke DNA methylation of tumor suppressor genes that may finally determine the onset of leukemia.

However, several works performed on police officers and gas station employers stated that low exposure to airborne benzene is correlated with modifications in DNA methylation in blood DNA of normal subjects that are similar to those discovered in hematological malignancies [148,149,150,151,152].

As reported above, exposure to benzene can act on *p14* and *p15* [147]. Deactivation of *p14ARF* would cause degradation of the p53 protein, a possible objective for methylation in tumor [153]. Increased methylation of *p15INK4b* would also provoke a five-fold rise in the risk of *p53* methylation [154]. Both genes are elements of the inhibitors of CDK4 (INK4) family of cyclin-dependent kinase (CDK) inhibitors positioned at the same chromosomal region. An augment of methylation abnormalities at their promoter region, which is the usual system for loss of tumor suppressor genes activity, is a possible mechanism able to cause hematologic malignancies [155].

In an in vivo study performed on a Bulgarian group of residents, gene-specific reduction of methylation of *p15INK4b* is described after exposure to benzene [156]. Moreover, hypermethylation of the p15 promoter, which undoubtedly participates in alteration of cell growth and is linked to AML, was reported in benzene-exposed subjects [157].

Moreover, exposure to benzene seems to be able of changing the expression of several other specific genes. Studies executed employing microarray analysis and real-time PCR demonstrated that the expression of several genes, *JUN*, *PF4*, *CXCL16*, *and ZNF331* were remarkably modified in shoe labors [158]. These findings were corroborated by a different research employing Affymetrix and Illumina platforms, and more DEGs were demonstrated to be implicated in programmed cell death [159]. Moreover, the authors successively evaluated total gene expression in peripheral blood mononuclear cells (PBMCs) from employers subjected to <1 ppm to >10 ppm of benzene. In addition to the AML and immune response pathways, a 16-gene expression mark was reported to be correlated with all benzene exposure amounts [160]. Schiffman and McHale evaluated 30 DEGs modified by benzene. They demonstrated that three pairs of genes implicated in immunologic response and inflammation, *ACSL1/CLEC5A*, *PRG2/CLEC5A*, and *NFKBI/CLEC5A*, were predictive of benzene contact at amounts below 1 ppm [161].

Three other hypermethylated genes with contemporaneous mRNA reduction (*PRKG1*, *PARD3*, *and EPHA8*) and two hypomethylated genes with augmented mRNA amount (*STAT3*, *IFNGR1*) were also recognized in benzene poisoning subjects [162]. Successive pathway evaluation recognized STAT3 as a main actor in numerous enriched carcinogenesis-relevant gene sets, comprising AML. Promoter DNA hypermethylation of the tumor suppressor genes p15 and p16 was reported in benzene-exposed employers, alongside with a reduction in the mRNA concentration [163]. However, an experimentation performed on gravid animals demonstrated that benzene exposure provoked a reduction of total methylation, but that p15 promoter methylation was unaffected in maternal BM cells and fetal livers, suggesting that this epigenetic effect to benzene exposure could be species-specific [164].

Furthermore, in a study executed employing rat BM cells, genes that regulate programmed cell death were explored [165]. Adding a DNA methyltransferase inhibitor to the benzene-exposed cells augmented the mRNA concentrations of Bcl-2-associated X protein (Bax) and Caspase-3 (Cas3), well-known apoptosis inhibitors, and reduced the amount of cell death in benzene-exposed BM cells. This indicates that benzene-induced cytotoxicity is modulated by epigenetic alteration of programmed cell death-blocking genes.

A reduction in the expression of PTEN, a tumor suppressor gene, and a relevant rise of the PTEN methylation amount was reported in animals exposed to benzene and in F32 human lymphoblast cells treated with benzene in a dose-dependent manner [166]. Expression of the repair gene PARP-1 was also reduced with promoter increased methylation in human lymphoblastoid F32 cells exposed to 10 mM benzene [167].

Finally, a particular epigenetic mechanism exerted by benzene on the induction of hematological neoplasms could be the alteration of the expression of the non-coding genetic material, a group of regulatory RNAs with huge significance in numerous diseases such as allergic, auto immune, and neoplastic disorders [168].

In a report, a total of six miRNAs were increased (let-7d, miR-10b, miR-34a, miR-205, and miR-423-2-5p) and seven decreased (miR-27b, miR-130a, miR-133a, miR-142-5p, miR-185, miR-223, miR-320b, and miR-543) in the blood of subjects with chronic benzene exposure with respect to normal subjects [169]. However, in a different study a correlation between benzene exposure and abnormal miRNA expression was also described in a non-occupational group of subjects. miR-223 expression in gravid women and indoor levels of benzene and toluene were positively correlated and seemed to reduce the number of regulatory T-cells in maternal and cord blood [170]. Animals that were treated with benzene for 4 weeks displayed relevant hematotoxicity, as well as modifications of numerous miRNAs in the BM cells of treated animals. Five miRNAs were augmented, and 45 miRNAs were reduced [171]. The augmented miRNAs were miR-34a-5p, miR-129b-3p, miR-129b-5p, miR-144-5p, and miR-451a, and the most greatly reduced miRNAs were let-7i-3p, miR-33-5p, miR-128-1-5p, miR-188-5p, miR-211-5p, miR-224-5p, miR-504-5p, miR-5107-3p, and miR-5120.

Furthermore, in an experimentation performed on benzene-exposed employers, the expression of two long noncoding (lnc)RNAs (lnc 028291 and lnc 045623) was greater in the samples of exposed employers with respect to controls [172]. These lncRNAs and their correlated mRNAs are implicated in hematopoiesis, chronic myeloid leukemia, immune response, and B cell receptor signaling, indicating their correlation with benzene-caused leukemogenesis.

In conclusion, long-term exposure to benzene appears to be capable of modifying the epigenetic pattern. Genes involved in the inhibition of proliferation, in apoptosis, in the immune response and in inflammatory processes can be differently expressed after exposure to benzene. This could be an essential moment in the carcinogenic process.

## 5. Future Perspectives

Epigenetic silencing of tumor suppressor genes through DNA increased methylation has been recognized as a common signature of oncogenesis [173]. However, the discovery of the systems of epigenetic induction in the onset of benzene-caused hematological tumors has allowed the possibility to operate with pharmacological interventions able of stopping or overturning the negative effects of benzene.

The methylation of CpG dinucleotides, especially at gene promoters and regulatory regions, has been demonstrated to cause epigenetic gene silencing through the employment of methyl-binding domain (MBD) proteins such as Methyl-CpG Binding Domain Protein 1 (MBD1), MBD2, and Methyl-CpG Binding Protein 2 (MeCP2) and their correlated chromatin remodeling/co-repressor complexes such as Mi2-NuRD [174]. These compounds are able of modifying the chromatin status, provoking a transcriptional repression [175]. Current pharmacological attempts for blocking of epigenetic gene silencing in tumors were directed on blocking DNMTs, and histone deacetylases (HDACs) [176]. Remarkably, such an attempt was conducted according to FDA authorization of two DNMT inhibitors and two HDAC inhibitors for the treatment of MDS and cutaneous T-cell lymphoma [177,178,179].

In the next future, other different therapeutic approaches seem possible. The bromodomain (BrD) operates as the acetyl-lysine binding domain to control gene stimulation in chromatin [180,181]. The human bromodomains are divided into subgroups with different characteristics [182]. One main group is BET (bromodomain and extra-terminal domain) proteins comprised of BRD2, BRD3, BRD4, and BRDT [183]. BRD4, perhaps the most extensively widely studied BET protein has a central role in several pathologies comprising tumors [184,185,186]. Unfortunately, in spite of the discovery of numerous powerful BET inhibitors, there is still no inhibitor able to differentiate between the different bromodomains within any specific BET protein. Discovering such a selective inhibitor is a promising possibility.

However, this is a difficult mission, as is determining the sequence identity of these bromodomains, especially at their acetyl lysine binding pockets [187,188]. Zhang et al. described the structure-guided generation of a novel group of diazobenzene based small molecule blockers for the BET bromodomains. MS436 is the best inhibitor produced employing a structure-activity relationship analysis [189]. They enhanced the affinity of the diazobenzene compounds toward the BRD4 BrD1 by over 100-fold. MS436 has an evaluated *Ki* of 30–50 nM for the BRD4 BrD1 with a 10-fold selectivity over the BrD2. This could assign to this substance drug-like capacities. Moreover, they verified the effectiveness of four lead diazobenzene BrD inhibitors in blocking BRD4 transcriptional activity in LPS-stimulated, NK-κB-directed production of nitric oxide and IL-6 in murine macrophage RAW264.7 cells [189].

Finally, in an investigation to find DNMT inhibitors from Formosan plants, Weng et al. recognized kazinol Q {4-[6-(1,1-dimethyl-allyl)-7-hydroxy-chroman-2-yl]-3,6-bis-(3-methyl-but-2-enyl)-benzene-1,2-diol} as an inhibitor of recombinant DNMT1 with IC50 of 7 mM [190]. The efficacy of kazinol Q on DNMT block was confirmed by its capacity to restart the expression of a DNA methylation-silenced gene, E-cadherin, in MDA-MB-231 tumoral cells. Furthermore, kazinol Q reduced the growth of tumor cells via the stimulation of programmed cell death. The action of DNMT1 inhibition in determining kazinol Q’s antiproliferative effect was confirmed by the protective action of ectopic expression of DNMT1 on kazinol Q-caused cell death. Molecular modeling evaluation proposes that kazinol Q blocks DNMT action by contending with cytosine binding, a system analogous to that reported for (-)-epigallocatechin-3-gallate (EGCG). However, in comparison to EGCG, kazinol Q shows numerous advantageous characteristics, comprising chemical stability and augmented hydrophobicity, and might have therapeutic application to leukemia treatment [190].

## 6. Conclusions

It is logical to assume that benzene and its metabolites exert different actions on different elements of the hematopoietic system able to provoke haemato-toxicity and malignancy. The possible mechanisms conducting to the onset and progression of the hematologic malignancies are described as a “multi-hit” model. Cancer stem-like cells may result from tumorigenic modifications commenced in normal, self-renewing hemopoietic stem cells or subsequent progenitors by benzene metabolites, causing the proliferation of the stem cell and progenitor pools and production of preleukemic stem cells. Secondary events may happen in the proliferating cells generating cancer stem like cells. Genomic instability and changes of cellular phenotypes, such as epigenetic alterations, may also happen. Benzene induced epigenetic alterations could operate on nuclear receptors, and provoke post-translational alterations at the protein level, so touching the stability or function of regulatory proteins, comprising oncoproteins and tumor suppressor proteins [191].

A further study of the epigenetic mechanisms of benzene-induced hematological alterations could open the way to new therapeutic possibilities.

## Figures and Tables

**Table 1:** Epigenetics alterations in hematological malignancies.

Disease	Status	Target	Type of Study	Ref.
AML	Hypomethylation	MAGE-1	In vitro	[122,123]
AML (HLA60 cell line)	Hypermethylation	H3K4mc3	In vitro	[138]
ALL (TK6 cells lymphoblastoid cells)	Hypomethylation	DNA	In vitro	[140]
ALL (Children)	Hypermethylation	MLL-r	Ex vivo	[132]
Experimental model		Target	Type of study	Ref.
Male C76B/6 mice		Leukemia stem cell quiescence and self renewal genes	In vivo	[135]
Exposed subjects	Hypomethylation	p15, MAGE-1, Line-1	Ex vivo	[136]
Exposed subjects	Hypomethylation	P15INK4b	Ex vivo	[157]
Exposed subjects		JUN, PF4, CXCL16, ZNF331	Ex vivo	[158]
Exposed subjects	Hypomethylation Hypomethylation	PRKG1, PARD3, EPHAS. STAT3, IFNGR1	Ex vivo	[162]
Exposed subjects	Hypermethylation	p15, p16	Ex vivo	[163]
Bone marrow rat cells, F32 lymphoblast cells	Hypermethylation	PTEN	In vitro and in vivo	[166]
